# Exploring the Impact of Gender on the Characteristics and Complications of Eosinophilic Esophagitis

**DOI:** 10.1002/jgh3.70059

**Published:** 2024-12-20

**Authors:** Het Patel, Joshua Elmer, Hammad Liaquat

**Affiliations:** ^1^ St. Luke's University Healthcare Network Bethlehem, PA USA

**Keywords:** eosinophilic esophagitis, esophageal impaction, food bolus, gender

## Abstract

**Background:**

Eosinophilic esophagitis (EoE) is a chronic inflammatory process of the esophagus often associated with structural and motility problems. Previous studies have shown an increased prevalence in males over females, however there is little data exploring the risk of esophageal complications among genders, which may be indicative of differences in disease severity.

**Methods:**

This is a retrospective cohort study using National Inpatient Sample data including adults hospitalized between 2016 and 2020 presenting with EoE. The primary outcome measured was inpatient complications related to the patient's history of EoE and secondary outcomes include demographics, comorbidities, month of presentation, and age of patients.

**Results:**

Of the 21 755 patients with history of EoE, 112 260 (52%) were male and 10 495 (48%) were female. Males had higher rates of several EoE complications, including esophageal obstruction, food bolus, esophageal rupture, requiring esophageal dilation, requiring esophageal laceration repair with *p* < 0.05 for all. Higher rates of females with EoE were seen in the Black population (*p* < 0.05). We also found that males were most likely to have esophageal impaction and esophageal rupture in the month of March (*p* < 0.05). Esophageal impaction was more common in males ages 25–29 (*p* < 0.05), whereas females were more likely from age 75–79 (*p* < 0.05).

**Conclusion:**

Males have an overall high rate of complication from EoE. There was a higher prevalence of females with EoE in the Black population although the rates of complication remained higher in males in this subpopulation. The month of March carries a risk of esophageal impaction and rupture pronounced particularly in men. Age also appears to have an influence on the rate of esophageal impaction.

## 1 Introduction

Eosinophilic esophagitis was first identified in the 1970s, and it was not until the 1990s that the disease became established in medical literature [1, 2, 3, 4]. Eosinophilic esophagitis is described as a chronic eosinophil predominant inflammation of the esophagus resulting in hyperplasia of the basal epithelial layer, dilated intracellular spaces, and thickening of the lamina propria causing remodeling that also impairs esophageal motor function [5, 6, 7]. It has been well established that the disease has a strong male predilection; however, little data exists further exploring these gender differences and understanding why they exist. The general consensus in the literature describes EoE to be two to three times more likely to present in males [8, 9, 10, 11].

It is widely accepted that EoE is caused by an allergic process that triggers a Th2 inflammatory response as about 70% of patients have a history of allergic disease, symptoms can be exacerbated by specific allergens, and multiple pro‐inflammatory markers have been identified to be associated with EoE [12, 13, 14]. Gender differences in Th2 inflammatory responses are well‐known to exist, however, as in conditions such as atopic dermatitis, females tend to have a higher prevalence explained by a more robust Th2 response [15]. The Th2 inflammatory cascade consists of many cytokines that cause this disease process and may account for these observed gender differences. One of the initial cytokines released by esophageal epithelial and dendritic cells in response to a stimulus is thymic stromal lymphopoietin (TSLP). Importantly, genetic mutations in this cytokine have been linked to increased susceptibility to EoE even without allergic history [16, 17, 18, 19]. Although the gene for this cytokine is autosomal, the TSLP receptor gene, CRLF2, is located on the pseudoautosomal region of the sex chromosomes and may be one possible explanation for the male prevalence of EoE [16].

Because of the paucity of studies assessing EoE between sexes in a large patient population, we conducted a retrospective analysis using the National Inpatient Sample (NIS) to assess the differences in complications between hospitalized males and females with new or previously diagnosed EoE. Given the greater male prevalence in EoE and multiple theories to explain this disparity, we hypothesized that males are more prone to the complications of EoE which may be due to males also suffer from a greater disease severity.

## 2 Methods

This is a retrospective cohort study using the NIS developed for the Healthcare Cost and Utilization Project (HCUP). The HCUP is a publicly available database that approximates a 20% sample of all discharges from US hospitals. The NIS is de‐identified and does not include any individualized patient data and therefore does not require institutional review board permission.

We included patients 18 years and older admitted from 2016 to 2020 with previously or newly diagnosed eosinophilic esophagitis. Diagnoses and procedures were identified using International Classification of Diseases, Tenth Revision, Clinical Modification (ICD 10 CM) codes and International Classification of Diseases, Tenth Revision, Procedure Coding System (ICD 10 PCS) codes, respectively. Variables such as age, race, mortality, month of presentation, and Charlson comorbidity index (CCI) were included in the NIS database. Other diagnoses including diabetes, hypertension, congestive heart failure (CHF), cerebrovascular disease, alcohol use disorder, substance use disorder, cancer, dementia, and obesity were available in the NIS database through the CCI and Elixhauser comorbidity index. The ICD 10 codes used to identify each diagnosis and procedure are outlined in the Data S1.

The primary outcomes compared were differences in inpatient complications of EoE in males versus females. Secondary variables assessed include demographics, comorbidities, month of presentation, and age of patients. Statistical analysis was conducted using STATA 17.0 to perform Chi Square testing and univariate analysis to calculate unadjusted odds ratios (OR). A cutoff of *p* < 0.05 was deemed to be statistically significant.

## 3 Results

Demographic data from 21 755 total cases of EoE is reported in Table 1. A total of 11 260 (52%) of EoE cases were males. The average age of females was 2.84 years older than male patients (*p* < 0.001). Of our sample, 79% of all patients were White with more males (80.4%) compared with females (76.9%) (*p* = 0.006, OR = 1.232; 95% CI 1.063–1.429). A lower percentage of male patients (6.4%) were Black compared with females (9.3%) (*p* < 0.001, OR = 0.668; 95% CI 0.529–0.843). Obesity was higher in the female population (11.3% in males and 18.3% in females) (p < 0.001, OR = 0.567; 95% CI 0.477–0.674). Men also had lower rates of diabetes, 11.9% versus 14.2% (*p* = 0.028, OR = 0.821; 95% CI 0.688–0.979). More men had alcohol use disorder (9.1% versus 4.8%) (OR = 1.972; 95% CI 1.537–2.530). Rates of hypertension, congestive heart failure, chronic obstructive pulmonary disease, end‐stage renal disease, cerebrovascular disease, cancer, dementia, malnutrition, and cirrhosis were not significantly different between male and female cases. In analyzing patients' CCI, we found a statistically significant greater number of males with a CCI of 0 (*p* = 0.001, OR = 1.220; 95% Cl 1.080–1.379) and more females with CCI of 1 (*p* = 0.004 OR = 0.824 95% CI 0.723–0.940) with higher CCI not being significantly different (*p* > 0.05) between genders.

**TABLE 1:** Demographics and comorbidities.

	Eosinophilic esophagitis in males (*n* = 11 260)	Eosinophilic esophagitis in females (*n* = 10 495)	*p*	Odds ratio (95% confidence interval)
Mean age, years	46.100	48.940	< 0.001	—
Race, *n*				
White	9055 (80.4%)	8075 (76.9%)	0.006	1.232 (1.063–1.429)
Black	720 (6.4%)	975 (9.3%)	< 0.001	0.668 (0.529–0.843)
Hispanic	704 (6.3%)	615 (5.9%)	0.575	1.074 (0.837–1.379)
Asian or Pacific Islander	115 (1.0%)	160 (1.5%)	0.158	0.667 (0.379–1.175)
Native American	25 (0.2%)	20 (0.2%)	0.818	1.167 (0.313–4.348)
Other	270 (2.4%)	235 (2.2%)	0.719	1.074 (0.729–1.582)
Obesity, *n*	1270 (11.3%)	1925 (18.3%)	< 0.001	0.567 (0.477–0.674)
Diabetes, *n*	1345 (11.9%)	1490 (14.2%)	0.028	0.821 (0.688–0.979)
Hypertension, *n*	3330 (29.6%)	2865 (27.3%)	0.098	1.120 (0.979–1.280)
Congestive heart Failure, *n*	700 (6.2%)	790 (7.5%)	0.095	0.815 (0.641–1.037)
COPD, *n*	730 (6.5%)	675 (6.4%)	0.938	1.010 (0.794–1.284)
ESRD, *n*	165 (1.5%)	200 (1.9%)	0.272	0.766 (0.476–1.234)
Cerebrovascular disease, *n*	340 (3.0%)	285 (2.7%)	0.549	1.117 (0.778–1.602)
Alcohol use disorder, *n*	1020 (9.1%)	500 (4.8%)	< 0.001	1.972 (1.537–2.530)
Substance use disorder, *n*	690 (6.1%)	550 (5.2%)	0.204	1.182 (0.913–1.539)
Cancer, *n*	450 (4.0%)	315 (3.0%)	0.083	1.347 (0.961–1.888)
Dementia, *n*	170 (1.5%)	165 (1.6%)	0.871	0.961 (0.592–1.559)
Malnutrition, *n*	490 (4.4%)	510 (4.9%)	0.441	0.892 (0.667–1.194)
Cirrhosis, *n*	295 (2.6%)	215 (2.0%)	0.218	1.288 (0.860–1.923)
Charlson comorbidity index				
0	4805 (42.7%)	3970 (37.8%)	0.001	1.220 (1.080–1.379)
1	3135 (27.8%)	3350 (31.9%)	0.004	0.824 (0.723–0.940)
2	1475 (13.1%)	1440 (13.7%)	0.562	0.949 (0.795–1.133)
3	735 (6.5%)	625 (6.0%)	0.429	1.104 (0.864–1.410)
4	460 (4.1%)	530 (5.1%)	0.129	0.802 (0.602–1.067)
5 or more	650 (5.8%)	580 (5.5%)	0.721	1.048 (0.809–1.358)

Rates of complications associated with the reported hospitalization are reported in Table 2. Males had higher rates of several EoE complications, including esophageal obstruction (17.1% vs. 11.9%; *p* < 0.001, OR = 1.529; 95% CI 1.529–1.819), food bolus (10.3% vs. 3.9%; *p* < 0.001, OR = 2.841; 95% CI 2.186–3.693), esophageal rupture (4.9% vs. 1.2%; *p* < 0.001, OR = 4.098; 95% CI 2.657–6.322), requiring esophageal stricture dilation (1.1% vs. 0.4%; *p* = 0.013, OR = 2.818; 95% CI 1.205–6.591), and requiring esophageal laceration repair (0.6% vs. < 0.1%; *p* = 0.002, OR = 12.193; 95% CI 1.594–93.275).

**TABLE 2:** Hospitalization complications.

	Eosinophilic esophagitis in males (*n* = 11 260)	Eosinophilic esophagitis in females (*n* = 10 495)	*p*	Odds ratio (95% confidence interval)
Esophageal dilation, *n*	120 (1.1%)	40 (0.4%)	0.013	2.818 (1.205–6.591)
Esophageal laceration repair, *n*	65 (0.6%)	5 (< 0.1%)	0.002	12.193 (1.594–93.275)
Esophageal obstruction/stricture, *n*	1920 (17.1%)	1245 (11.9%)	< 0.001	1.529 (1.529–1.819)
Food impaction, *n*	1165 (10.3%)	410 (3.9%)	< 0.001	2.841 (2.186–3.693)
Esophageal rupture, *n*	550 (4.9%)	130 (1.2%)	< 0.001	4.098 (2.657–6.322)
Esophageal hemorrhage, *n*	25 (0.2%)	15 (0.1%)	0.542	1.556 (0.372–6.518)
Esophageal perforation, *n*	110 (1.0%)	25 (0.2%)	0.002	4.136 (1.563–10.945)

Rates of EoE related complications were further assessed in a subgroup analysis reported in Table 3. Despite higher number of females in the Black population, the rates of esophageal dilation (2.8% vs. 0.0%; *p* = 0.006, OR = 4.266; 95% CI 1.376–13.231) and esophageal obstruction (20.8% vs. 18.0%; *p* = 0.036, OR = 1.574; 95% CI 1.026–2.415) were higher in males. The statistically significant gender differences seen in rates of esophageal laceration repair, food impaction, esophageal rupture, and esophageal perforation seen in the total sample was not seen in the Black population (*p* > 0.05). Esophageal hemorrhage was also not significant (*p* < 0.05) in the Black subgroup analysis.

**TABLE 3:** Hospitalization complications in Black patients.

	Eosinophilic esophagitis in males (*n* = 720)	Eosinophilic esophagitis in females (*n* = 975)	*p*	Odds ratio (95% confidence interval)
Esophageal dilation, *n*	20 (2.8%)	0 (0.0%)	0.006	4.266 (1.376–13.231)
Esophageal laceration repair, *n*	5 (0.7%)	0 (0.0%)	0.411	2.257 (0.307–16.570)
Esophageal obstruction/stricture, *n*	150 (20.8%)	175 (18.0%)	0.036	1.574 (1.026–2.415)
Food impaction, *n*	50 (6.9%)	35 (3.6%)	0.890	0.955 (0.493–1.831)
Esophageal rupture, *n*	10 (1.4%)	0 (0.0%)	0.225	0.428 (0.104–1.756)
Esophageal hemorrhage, *n*	5 (0.7%)	5 (0.5%)	0.147	4.198 (0.511–34.498)
Esophageal perforation, *n*	5 (0.7%)	0 (0.0%)	0.908	1.125 (0.151–8.368)

No statistically significant difference was noted in the rates of EoE hospitalizations in males versus females in each month (*p* > 0.05): January (7.5% vs. 6.6%), February (7.6% vs. 8.7%), March (7.5% vs. 9.0%), April (7.5% vs. 8.1%), May (8.6% vs. 7.5%), June (9.2% vs. 8.2%), July (8.6% vs. 9.1%), August (9.1% vs. 8.5%), September (8.5% vs. 9.3%), October (8.0% vs. 9.0%), November (9.3% vs. 7.9%), and December (8.3% vs. 8.1%).

Rates of specific complications including esophageal rupture and esophageal impaction between sexes by month of the year are reported in Table 4. Males had higher rates of esophageal rupture than females in 5 months of the year: March (3.3% vs. 0.3%; *p* = 0.001, OR = 14.446; 95% CI 1.857–112.350), April (2.9% vs. 0.9%; *p* = 0.047, OR = 3.501; CI 0.945–12.977), July (2.9% vs. 0.5%; *p* = 0.012; OR = 5.680; 95% CI 1.244–25.943), November (2.7% vs. 0.3%; *p* = 0.018, OR = 8.250; 95% CI 1.046–65.072), and December (2.8% vs. 0.6%; *p* = 0.030, OR = 4.746; 95% CI 1.016–22.176). Additionally, esophageal impaction rates in males were significantly higher (*p* < 0.05) for the majority of the year: January, March, April, May, June, July, October, November, and December.

**TABLE 4:** Esophageal rupture and impaction stratified by month.

	Esophageal rupture			Esophageal impaction		
	Male/female	*p*	Odds ratio (95% confidence interval)	Male/female	*p*	Odds ratio (95% confidence interval)
January, (*n* = 1535)	1.0%/0.0%	0.113	—	5.2%/1.0%	0.008	4.706 (1.343–16.486)
February, (*n* = 1775)	2.3%/0.8%	0.101	2.927 (0.765–11.198)	3.1%/1.1%	0.050	3.057 (0.947–9.874)
March, (*n* = 1795)	3.3%/0.3%	< 0.001	14.446 (1.857–112.350)	3.6%/0.6%	0.002	7.833 (1.742–35.229)
April, (*n* = 1695)	2.9%/0.9%	0.047	3.501 (0.945–12.977)	6.5%/1.8%	0.002	4.091 (1.605–10.427)
May, (*n* = 1750)	2.6%/0.6%	0.071	3.791 (0.807–17.810)	6.9%/2.0%	0.009	3.043 (1.282–7.226)
June, (*n* = 1900)	3.4%/1.1%	0.063	2.831 (0.902–8.886)	5.8%/2.1%	0.032	2.453 (1.054–5.708)
July, (*n* = 1925)	2.9%/0.5%	0.012	5.680 (1.244–25.943)	6.2%/1.3%	< 0.001	5.252 (1.965–14.039)
August, (*n* = 1920)	2.1%/0.5%	0.090	3.556 (0.745–16.959)	3.1%/2.1%	0.099	2.035 (0.862–4.800)
September, (*n* = 1935)	2.3%/1.0%	0.150	2.348 (0.710–7.764)	3.1%/3.6%	0.715	0.862 (0.387–1.918)
October, (*n* = 1840)	1.9%/0.5%	0.080	3.763 (0.771–18.368)	6.3%/2.2%	0.003	3.296 (1.433–7.580)
November, (*n* = 1880)	2.7%/0.3%	0.017	8.250 (1.046–65.072)	6.6%/2.1%	0.016	2.669 (1.170–6.089)
December, (*n* = 1785)	2.8%/0.6%	0.030	4.746 (1.016–22.176)	6.2%/2.5%	0.031	2.385 (1.064–5.347)

Rates of EoE cases between sexes were stratified by age group of hospitalization in Table 5 and subsequently analyzed separately for rates of esophageal impaction and esophageal rupture. For all EoE cases, males had higher rates of EoE hospitalizations for patients ages 20–24 years old (9.7% vs. 6.5%; *p* < 0.001; OR = 1.536; 95% CI 1.226–1.925). However, males had lower rates of EoE hospitalizations in older age groups: 70–74 (4.3% vs. 6.1%; *p* = 0.009, OR = 0.694; 95% CI 0.528–0.912), 80–84 (2.3% vs. 3.9%; *p* = 0.004, OR = 0.589; 95% CI 0.412–0.843), and 85–89‐year‐old (0.9% vs. 2.2%; *p* = 0.001, OR = 0.421; 95% CI 0.250–0.707). Rates of esophageal impaction and esophageal rupture were then compared between sexes and stratified by age group. A total of 15.5% of male cases of esophageal impaction were between ages 25–29 while 4.9% of female cases were in this age group (*p* = 0.014, OR = 3.564; 95% CI 1.221–10.395). Female cases of esophageal impaction were significantly higher in older patients ages 75–79 (1.7% of male versus 7.3% of female) (*p* = 0.013, OR = 0.221; 95% CI 0.061–0.806).

**TABLE 5:** EoE total, esophageal impaction, esophageal rupture age stratified.

	Total			Esophageal impaction			Esophageal rupture		
	Males/female (11 260/10495)	*p*	Odds ratio (95% confidence interval)	Males/female (1165/410)	*p*	Odds ratio (95% confidence interval)	Males/female (550/130)	*p*	Odds ratio (95% confidence interval)
20–24, *n*	9.7%/6.5%	< 0.001	1.536 (1.226–1.925)	11.2%/6.1%	0.183	1.934 (0.724–5.168)	12.7%/3.8%	0.194	3.646 (0.457–29.092)
25–29, *n*	8.0%/7.0%	0.226	1.155 (0.915–1.458)	15.5%/4.9%	0.014	3.564 (1.221–10.395)	13.6%/11.5%	0.776	1.211 (0.325–4.510)
30–35, *n*	8.9%/9.1%	0.778	0.969 (0.779–1.206)	10.3%/8.5%	0.646	1.230 (0.507–2.985)	17.3%/11.5%	0.475	1.601 (0.436–5.879)
35–39, *n*	9.3%/9.3%	0.997	1.000 (0.812–1.231)	13.3%/6.1%	0.079	2.363 (0.883–6.322)	12.7%/15.4%	0.719	0.802 (0.240–2.675)
40–44, *n*	8.3%/8.1%	0.810	1.029 (0.817–1.295)	7.3%/6.1%	0.716	1.212 (0.430–3.417)	7.3%/7.7%	0.942	0.941 (0.186–4.752)
45–49, *n*	8.0%/7.9%	0.823	1.025 (0.822–1.279)	9.4%/14.6%	0.203	0.608 (0.281–1.315)	10.0%/11.5%	0.817	0.852 (0.219–3.306)
50–55, *n*	8.8%/8.0%	0.356	1.108 (0.891–1.378)	9.9%/11.0%	0.777	0.888 (0.391–2.019)	10.0%/15.4%	0.430	0.611 (0.178–2.098)
55–59, *n*	8.5%/8.6%	0.918	0.989 (0.795–1.228)	6.0%/9.8%	0.254	0.591 (0.238–1.470)	5.5%/3.8%	0.738	1.442 (0.167–12.490)
60–64, *n*	7.5%/6.4%	0.145	1.199 (0.939–1.529)	6.0%/9.8%	0.245	0.591 (0.242–1.445)	4.5%/3.8%	0.880	1.190 (0.124–11.433)
65–69, *n*	6.0%/7.0%	0.171	0.848 (0.669–1.074)	4.3%/4.9%	0.825	0.875 (0.267–2.867)	1.8%/0.0%	0.486	—
70–74, *n*	4.3%/6.1%	0.009	0.694 (0.528–0.912)	2.1%/3.7%	0.452	0.577 (0.136–2.446)	0.0%/3.8%	0.038	—
75–79, *n*	3.4%/4.3%	0.120	0.782 (0.573–1.067)	1.7%/7.3%	0.013	0.221 (0.061–0.806)	2.7%/3.8%	0.761	0.701 (0.070–7.029)
80–84, *n*	2.3%/3.9%	0.004	0.589 (0.412–0.843)	0.0%/1.2%	0.091	—	0.0%/0.0%	—	—
85–89, *n*	0.9%/2.2%	< 0.001	0.421 (0.250–0.707)	0.4%/0.0%	0.553	—	0.0%/3.8%	0.038	—
90–94, *n*	0.8%/0.7%	0.746	1.120 (0.563–2.228)	0.4%/2.4%	0.107	0.172 (0.154–1.928)	0.0%/0.0%	—	—

Data on rates of comorbid conditions in males versus females with EoE are reported in Table 6. Notably, fewer male patients had asthma than female patients in this study (17.5% vs. 25.3%; *p* < 0.001, OR = 0.627; 95% CI 0.540–0.728). Similarly, rates of celiac disease were lower in male patients than female patients (0.8% vs. 1.6%; *p* = 0.21, OR = 0.517; 95% CI 0.292–0.916). However, men did have higher rates of achalasia than women (1.5% vs. 0.8%; *p* = 0.034, OR = 1.938; 95% CI 1.039–3.616). Rates of atopic dermatitis, multiple sclerosis, hypertrophic cardiomyopathy, ulcerative colitis, esophageal atresia, Crohn's disease, graft‐vs‐host disease, eosinophilic gastritis, and eosinophilic colitis did not demonstrate a statistically significant difference between men and women in this dataset (*p* > 0.05).

**TABLE 6:** EoE comorbidities.

	Eosinophilic esophagitis in males (*n* = 11 260)	Eosinophilic esophagitis in females (*n* = 10 495)	*p*	Odds ratio (95% confidence interval)
Atopic dermatitis, *n*	25 (0.2%)	30 (0.3%)	0.675	0.777 (0.238–2.540)
Asthma, *n*	1970 (17.5%)	2655 (25.3%)	< 0.001	0.627 (0.540–0.728)
Multiple sclerosis, *n*	40 (0.4%)	75 (0.7%)	0.103	0.496 (0.210–1.171)
Hypertrophic cardiomyopathy, *n*	15 (0.1%)	20 (0.2%)	0.638	0.699 (0.156–3.132)
Ulcerative colitis, *n*	210 (1.9%)	140 (1.3%)	0.162	1.407 (0.870–2.275)
Crohn's disease, *n*	300 (2.7%)	210 (2.0%)	0.158	1.342 (0.891–2.022)
Celiac disease, *n*	95 (0.8%)	170 (1.6%)	0.021	0.517 (0.292–0.916)
Esophageal atresia, *n*	5 (< 0.1%)	0 (0.0%)	0.334	—
Achalasia, *n*	165 (1.5%)	80 (0.8%)	0.034	1.938 (1.039–3.616)
Graft‐vs‐host disease, *n*	15 (0.1%)	5 (< 0.1%)	0.352	2.801 (0.291–27.001)
Eosinophilic gastritis, *n*	150 (1.3%)	190 (1.8%)	0.230	0.733 (0.441–1.219)
Eosinophilic colitis, *n*	30 (0.3%)	35 (0.3%)	0.687	0.799 (0.268–2.380)

## 4 Discussion

Eosinophilic esophagitis has a prevalence of 42 in 100 000 adults globally and has been reported in all age groups, with most cases found in Caucasian patients in the third and fourth decades of their lives [6, 8, 10, 20]. Age has not been shown to be associated with peak mucosal eosinophil count, but it has been suggested that fibrostenotic findings in EoE are significantly increased with age and are thought to be a consequence of ongoing progressive inflammation [10, 21, 22]. In evaluating rates of esophageal impaction based on age and gender, our results show that there is a significantly higher rate of esophageal impaction in males ages 25–29 and a high rate of impaction females ages 70–74. This is similar to other allergic diseases such as asthma and vernal keratoconjunctivitis, which are noted to improve with age in males, potentially due androgens having a protective immunosuppressive role although predominantly during puberty [23].

Although, as previously mentioned EoE is traditionally two to three times more prevalent in males, our study had roughly similar males and females (52% vs. 48%, respectively). This may be a selection bias as we included patients who presented to the hospital with EoE. They could also imply males are less likely to present inpatient and are potentially more responsive to outpatient treatment. Additionally, based on this potential underrepresentation, our data may not be applicable to all patients with EoE and be limited to those who present to the hospital as assessed by our data. Further studies may be useful to assess why these disparities exist.

Our data shows that males have a higher prevalence of EoE in the Caucasian population whereas females have a higher prevalence in the Black population which does not appear to have been described in previous literature to our knowledge. Based our literature review, it appears that many studies that describe a male predominance in EoE include a mostly Caucasian population. This difference may be due to Black females in fact being disproportionately affected but may also may have a social component with a higher rate Black females utilizing inpatient resources or being provided workup of symptoms or vice versa in males. In our subgroup analysis of our Black population, we continued to see similar male dominance in esophageal stricture and stricture dilation. However, there were no gender differences in esophageal laceration repair, food impaction, rupture, and hemorrhage. Besides for the higher male predilection for structuring, ethnicity may have significant impact on other esophageal complication in EoE.

Studies have also found *Helicobacter pylori* ( *H. pylori* ) infection to be inversely associated with EoE suggesting a possible protective effect of infection with this organism [24, 25, 26]. However, one study also found a statistically significant higher rate of EoE and *H. pylori* in males [25]. This may imply that while *H. pylori* has an impact on risk of developing EoE, it may not account for the gender disparity seen and further studies need to be conducted to evaluate this difference. Lymphocytic Esophagitis is a condition that presents similarly to EoE with its pathophysiology involving immune cell infiltration of the esophagus but does not carry the same male predominance, suggesting that the gender differences seen in EoE may be attributed to differences specific to the Th2 trigger or inflammatory response itself [27, 28, 29].

Multiple studies have also been performed to further investigate comorbidities associated with EoE. It is well established that history of a Th2 inflammatory disease such as asthma or IgE‐mediated allergy is strongly associated with EoE [24, 25, 26]. Based on the results, asthma appears to be a stronger risk factor in females than males as outlined in Table 6. The association between celiac disease and EoE has also been studied, however multiple large sample studies have not shown any association between gender groups [27, 28, 29, 30]. Our study however, showed females with celiac are statistically more likely to have EoE. EoE has also been associated with Crohn's disease, ulcerative colitis, multiple sclerosis, hypertrophic cardiomyopathy, esophageal atresia, graft‐versus‐host disease, and achalasia [5, 31, 32, 33, 34, 35, 36]. Besides for a significantly higher rate of achalasia in men with EoE, all other of these comorbid conditions did not show a gender preference. All results are consistent with known gender differences attributed to each comorbidity in Table 6 and we cannot say whether EoE has a confounding impact. Other eosinophil infiltrative gastrointestinal diseases such as eosinophilic gastritis and eosinophilic colitis do not share the same male prevalence as EoE.

One common complication of EoE is food impaction. About one‐third of patients with EoE experience at least one food bolus impaction that requires urgent endoscopic removal [37]. In fact, the rates of EoE reported are rising, a trend attributed to more providers performing esophageal biopsies after a food impaction [38, 39]. Impaction is thought to be the consequence of persistent eosinophilic inflammation which leads to deposition of subepithelial fibrous tissue and results in fibrostenotic structural and motility changes within the esophagus [40, 41, 42, 43, 44]. Males in general, at higher rates of esophageal obstruction and food bolus impaction. This may be attributable to males possibly having a more severe disease process or different manifestation of the disease compared to females. As mentioned earlier, males ages 25–29 and females ages 75–79 were more likely to have esophageal impaction compared to the opposite gender in the same age category. Furthermore, we found that males had a significantly higher rate of presenting to the hospital for esophageal impaction in March with the highest odds ratio of 7.833. Interestingly, we also found the rate of esophageal rupture in March to be far greater than that of females with an odds ratio 14.446. This may in part be to change in season during that time similar to other allergic processes but how this influences males disproportionately remains unclear. Furthermore, higher rates of complications were also seen in July, November, and December in both genders, which could be due to the holidays during these months which also may influence patients' diets. This may be an opportunity for intervention in the outpatient setting in educating patients to be more cautious when deviating from a patients staple diet particularly around these times.

Longstanding untreated EoE is a risk factor for esophageal strictures, with one study by Warners et al. [22] predicting that with each year of undiagnosed EoE, the risk of strictures increases by 9% [45]. This study also found that males with EoE were more prone to stricture formation and hypothesized that males are more prone to fibrosis due to lower estrogen levels, which may be responsible for counteracting the activity of myofibroblasts and fibrogenesis in women [22]. Previous studies have also described that males are more likely to present with food impaction, develop strictures, and complain of dysphagia symptoms [22, 45, 46, 47]. This was challenged by a Danish study of 236 patients that did not find any of these gender differences and attributed some of these findings to societal differences as most other EoE studies have been performed in the United States [48].

To prevent severe complications and symptoms, treatment is recommended for all patients with EoE. For those presenting with significant dysphagia symptoms or strictures, esophageal dilation may be an option. We found esophageal dilation be more likely to be performed in males which may be due to a higher rate of strictures in males. Although dilation has been shown to be effective and safe for symptom management in multiple studies including both men and women, it does not prevent or treat the underlying inflammatory disease process [49, 50, 51, 52, 53]. Furthermore, there are no guidelines for the timing of when dilation is most effective and carries the least complications, given that complications include perforation and rupture [54]. That being said, based on our study and findings of significantly higher rates of esophageal rupture and laceration repair in males, we recommend increased caution in male patients.

Proton pump inhibitors (PPIs) have been proposed to be effective in eosinophilic esophagitis not only for symptomatic relief by reducing the acidity of gastric juice, but it is found to inhibit IL‐4 stimulated eotaxin‐3 expression by blocking the gene promoter resulting in clinical, endoscopic, and histologic improvement in a certain population [55, 56]. The other mainstay of treatment is swallowed topical corticosteroid (STC) which has shown to be effective in achieving short‐term and long‐term endoscopic and histologic improvement and reduce the risk of bolus impaction [57, 58, 59, 60, 61, 62, 63]. Elimination diets and elemental diets have, similar to medical therapy, shown to be effective at improving EoE and helping to achieve remission in males and female patients [64, 65, 66, 67]. Finally, monoclonal antibody therapy is an emerging field that has shown very promising results in the treatment of EoE. Dupilumab is a well‐tolerated IL4 and IL13 antibody that has shown significant improvement in severe, refractory fibrostenotic disease [68, 69, 70].

Further studies are necessary to explore the gender differences in different ethnic groups given our data shows females have a higher prevalence in the Black community, and it is unclear what other differences are associated with ethnicity. Due to the limitations of the National Inpatient Sample in evaluating treatment, further studies are necessary exploring treatment response to PPI, STC, dietary changes, and monoclonal antibodies between males and females as this has not been explicitly reviewed in previous studies. Additionally, this database is limited in his ability to assess which patients have been admitted multiple times. Our study also does not take into consideration the primary reason for admission, which may influence analysis of complications as we assessed all patients admitted with EoE. Our data are also limited as our study employs univariate analysis which may not account for potential confounders.

In conclusion, it appears that males have an overall high rate of complication from EoE. We found a higher prevalence of females with EoE in the Black population although the rates of complication remained higher in males. Furthermore, the month of March carries a risk of esophageal impaction and rupture pronounced particularly in men. Age also appears to have an influence on the rate of esophageal impaction. Further studies assessing the pathophysiology and histologic manifestations are necessary determine if males indeed have a more severe disease process that accounts for higher rates of diagnosis of EoE in males.

## Conflicts of Interest

The authors declare no conflicts of interest.

## Supporting information


Data S1.
