# Supplementary material for: Exploring the Impact of Gender on the Characteristics and Complications of Eosinophilic Esophagitis
Source: JGH Open. 2024 Dec 20;8(12):e70059. doi: 10.1002/jgh3.70059 (PMC11660561; doi:10.1002/jgh3.70059)
Supplement: Supplementary file 1 — Data S1. [file JGH3-8-e70059-s001.docx]

| ICD Diagnosis and Procedure Codes | |
| --- | --- |
| COPD | J449, J441, J440 |
| ESRD | I120, N186, I132, Z992, N185, I1311 |
| Malnutrition | E441, E440, E46 |
| Cirrhosis | K745, K7469, K7030, K7031, K7460, K717, K744 |
| Eosinophilic Esophagitis | K200 |
| Esophagus Dilation | 0D754ZZ, 0D717DZ, 0D718DZ, 0D727DZ, 0D728DZ, 0D737DZ, 0D738DZ, 0D747DZ, 0D748DZ, 0D757DZ, 0D758DZ |
| Esophageal Laceration Repair | 0DQ50ZZ, 0DQ53ZZ, 0DQ54ZZ, 0DQ57ZZ, 0DQ58ZZ |
| Esophageal Obstruction/Stricture | K222 |
| Food Impaction | T18100A, T18108A, T18110A, T18118A, T18120A, T18128A, T18190A, T18198A |
| Esophagus Spontaneous Rupture | K223 |
| Esophagus Hemorrhage | K229 |
| Esophagus Perforation | S27819A, S27813A |
| Atopic Dermatitis | L200, L2081, L2082, L2083, L2084, L2089, L209 |
| Asthma | J45901, J45901, J4522, J4521, J4531, J4541, J4551, J4532, J4542, J4552, J45998, J8283, J45909, J45991, J4520, J4530, J4540, J4550, J45990 |
| Multiple Sclerosis | G35 |
| Hypertrophic Cardiomyopathy | I421, I422 |
| Ulcerative Colitis | K5180, K51913, K51914, K5190, K51911, K51912, K51919, K5150, K51813, K51814, K51812, K51818, K51918, K5100, K5120, K5130, K51318, K51319, K51019, K51219, K51314, K51014, K51214, K51011, K51211, K51311, K51313, K51013, K51213, K51012, K51212, K51312 |
| Crohn's Disease | K50919, K50911, K50912, K50913, K50914, K50112, K50011, K50013, K50014, K50111, K50113, K50114, K50119, K50012, K50812, K50019, K50811, K50813, K50814, K50819, K5010, K5000, K5080, K5090 |
| Celiac Disease | K900 |
| Esophageal Atresia | Q390, Q391 |
| Achalasia | K220 |
| Graft-vs-Host Disease | D89813, D89810, D89811, D89812 |
| Eosinophilic Gastritis | K5281 |
| Eosinophilic Colitis | K5282 |
